# Suicide and the agent–host–environment triad: leveraging surveillance sources to inform prevention

**DOI:** 10.1017/S003329172000536X

**Published:** 2021-03

**Authors:** Katherine M. Keyes, Sasikiran Kandula, Mark Olfson, Madelyn S. Gould, Gonzalo Martínez-Alés, Caroline Rutherford, Jeffrey Shaman

**Affiliations:** 1 Department of Epidemiology, Columbia University, New York, NY, USA; 2 Department of Environmental Health Sciences, Columbia University, New York, NY, USA; 3 Department of Psychiatry, Columbia University, New York, NY, USA; 4 Universidad Autónoma de Madrid School of Medicine, Madrid, Spain

**Keywords:** Suicide, contagion, environment, infectious, spatial, time trend, Google

## Abstract

Suicide in the US has increased in the last decade, across virtually every age and demographic group. Parallel increases have occurred in non-fatal self-harm as well. Research on suicide across the world has consistently demonstrated that suicide shares many properties with a communicable disease, including person-to-person transmission and point-source outbreaks. This essay illustrates the communicable nature of suicide through analogy to basic infectious disease principles, including evidence for transmission and vulnerability through the agent–host–environment triad. We describe how mathematical modeling, a suite of epidemiological methods, which the COVID-19 pandemic has brought into renewed focus, can and should be applied to suicide in order to understand the dynamics of transmission and to forecast emerging risk areas. We describe how new and innovative sources of data, including social media and search engine data, can be used to augment traditional suicide surveillance, as well as the opportunities and challenges for modeling suicide as a communicable disease process in an effort to guide clinical and public health suicide prevention efforts.

The increase in suicide, suicidal behavior, and mood disorders in the US demand a public health response. While global suicide mortality rates have decreased since the early 1980s (Naghavi, 2019), including significant reductions in China and India, suicide in the US has been increasing for approximately two decades, from 10.5 per 100 000 in 1999 to 14.2 per 100 000 in 2018 (Centers for Disease Control and Prevention, 2018a). Suicide deaths represent the ‘tip of the iceberg’ and data have documented increases in suicidal ideation and attempts, mood disorders, and psychological distress among US adolescents and adults. National, state, and local public health efforts are being mobilized to address this growing epidemic. Among the cascade of interventions that can be deployed to prevent suicide, high-quality surveillance to guide action is among the most basic public health needs. Existing surveillance efforts for suicide in the US are extensive (Crosby, Ortega, & Melanson, 2011), but few efforts have been made to assess the magnitude and location of spatial and temporal suicide correlations.

Suicides, and suicidal behavior, statistically cluster across time and space (Gould, Wallenstein, & Davidson, 1989). These clusters arise through multiple mechanisms mediated by seasonal, geographic, demographic, and meteorological factors, but also exhibit properties that resemble the spread of a contagion. Figure 1 shows the monthly suicides from 1999 to 2018. Suicide exhibits a seasonal trend, with increases in spring/summer months and decreases in winter months, an overall increase in the general trend across time, as well as some potentially anomalous increases at specific periods across the observed time series. All of these factors: trends, seasonality, anomalous changes, are often observed for contagious processes (Anderson, Grenfell, & May, 1984; Grassly & Fraser, 2006). This contagion hypothesis, that suicidal ideation can be shared, for example, by point-source and person-to-person transmission is supported by centuries of sociological and psychological literature on suicide, but has not been formally interrogated using communicable disease methods. While we know that outbreaks of suicide occur and that they are spatially and temporally correlated, less is understood about how many or what proportion of all suicide deaths arise from clusters, *v. de novo* (Gould, Wallenstein, & Kleinman, 1990; Insel & Gould, 2008). Outbreaks of suicide via person-to-person transmission typically affect a relatively small proportion of suicide decedents, and a larger proportion arises due to geographic and temporal autocorrelation (Sy et al., 2019), suggesting that clustering of suicide represents an important, although not complete, component of suicide etiology.

**Fig. 1.**
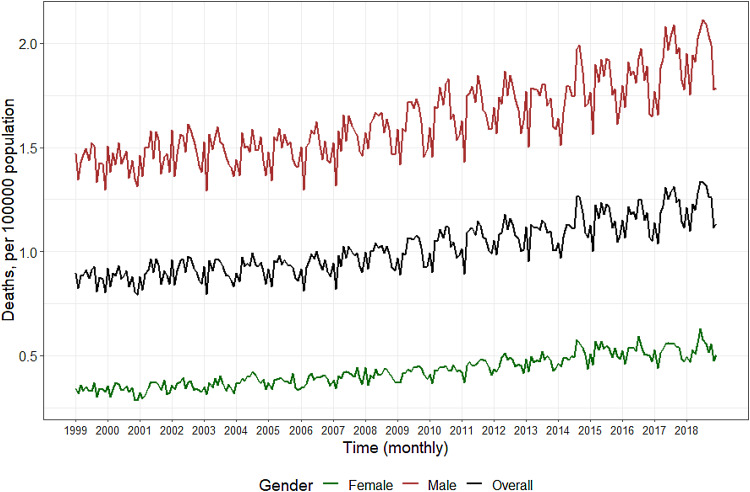
Monthly recorded deaths from suicide in the US, 1999–2018^1^.

The temporal and spatial features of suicide epidemiology indicate that suicide can and ought to be studied with the same tools with which we study other contagious and environmentally-mediated health outcomes. These various sources of time–space clustering can be conceptualized through the paradigm of the agent, host, and environment triad that underlies other communicable health outcomes. The agent–host–environment triad is a classic organizing framework for describing risk factors for infectious disease. Agent factors are the actual pathogens, such as bacteria and viruses, that cause the disease or the vectors that transmit them; host factors are individual-level factors that increase vulnerability to disease (e.g. older adults at increased risk for complications due to respiratory viruses); and environmental factors are those social and physical features that increase the risk for disease transmission or pathogenicity (e.g. some viruses are more transmittable in certain seasons). Similar to the classic agent–host–environment triad in infectious disease epidemiology, Figure 2 displays the hypothetical examples of the agent–host–environment triad for suicide risk. Public health surveillance aims to provide forward-looking information that anticipates where, when, and for whom the risk for suicide is concentrated and increasing. Such forecasts would be especially important when new risk factors occur, such as those associated with infectious disease epidemics or other natural disasters. Further, recognizing and modeling the ways in which suicide has communicable disease properties can complement clinical risk management, by providing tools that move beyond individual risk factors to aiding prediction of who is most at risk, when, and where.

**Fig. 2.**
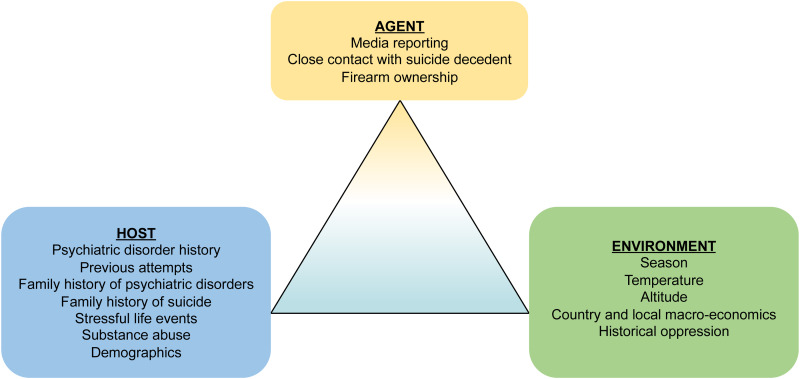
Epidemiological triad of communicable diseases, with examples from suicide.

The COVID-19 epidemic has placed mathematical models for understanding disease processes and forecasting disease progress in sharp focus across the world (Li et al., 2020), and emphasized the critical role of surveillance data, including how to appropriately use data that is incomplete (e.g. asymptomatic or undiagnosed cases). In this editorial, we describe the features of suicide that are analogous to other communicable diseases and provide an overview of the data that would be necessary to leverage existing methods to predict outbreaks and inform clinical care.

## Agent factors that are associated with suicide risk

Suicide can be transmitted similarly to a contagious process (Cheng, Li, Silenzio, & Caine, 2014; Haw, Hawton, Niedzwiedz, & Platt, 2013), including person-to-person transmission, as well as point-source outbreaks. Person-to-person transmission of suicide most commonly arises in smaller local suicide clusters (Cheng et al., 2014; Gould et al., 1989). These clusters occur primarily among adolescents and young adults in close geographic proximity (e.g. within a school or small community) (Gould et al., 1990, 2014). Psychological autopsy, ethnographic, and journalistic reporting suggest that suicide deaths within these clusters may have a direct causal relationship (Gould et al., 1989; Joiner, 1999), although these methods are insufficient alone for causal inference. Understanding the dynamics of these clusters is critical for identifying and estimating risk. Local clusters may arise in part due to processes of assortative social network formation; adolescents and young adults with shared vulnerabilities, including experiences of stressful life events, tend to form relationships, and it is this assortative tie formation and subsequent social learning that, in part, can create accumulated risk of suicidal behavior transmission (Gould et al., 1989). The spread of information and learning, coupled with the social network ties that concentrate risk, suggest that local suicide clusters share characteristics with person-to-person transmission of infectious diseases. Unlike infectious diseases, however, interventions to reduce suicide transmission are not provided in isolation, but rather to bolster supportive contacts within the social network and with providers. Indeed, theories of suicidal behavior arising from eusocial processes (Joiner, Hom, Hagan, & Silva, 2016) (perceived self-harm to reduce the burden on others, or for other sacrificial reasons) highlights the communicable aspect of suicidal behavior as existing within a social environment and strategies for its prevention.

Point-source outbreaks of suicidal behavior have been studied for centuries. Suicide rates increase with exposure to fictionalize and nonfiction suicide information, especially sensationalistic irresponsible media reporting, often in the wake of a high-profile suicide. Systematic and qualitative reviews (Sisask & Värnik, 2012; Stack, 2005) and meta-analysis (Niederkrotenthaler et al., 2012, 2020) indicate that suicide rates correlate with a number of media reports, nature of reporting, and demographic characteristics of the decedent and subsequent decedents. Increases in suicide following media reporting are often sudden and relatively short in duration. A recent example is the death of Robin Williams, in which the overall US suicide rate increased by >10% over the expected rate in the 5 months following his suicide (Fink, Santaella-Tenorio, & Keyes, 2018). Suicidal behavior exceeding expected patterns, which is consistent with, although not definitely demonstrating, a causal effect and has been observed across other countries (Pirkis et al., 2020a, 2020b; Whitley, Fink, Santaella-Tenorio, & Keyes, 2019). Netflix's show *13 Reasons Why* was also associated with anomalous increases in suicide death, especially among adolescent girls (Bridge et al., 2019; Niederkrotenthaler et al., 2019), although more data are needed. Conversely, responsible media reporting of suicide prevents contagion and spread of suicidal behavior (Niederkrotenthaler, 2016). The effect of media reporting on suicide is analogous to a rapidly and broadly distributed point-source outbreak of infectious disease. In the case of suicide, the ‘source’ of the outbreak is media reporting.

**Fig. 3.**
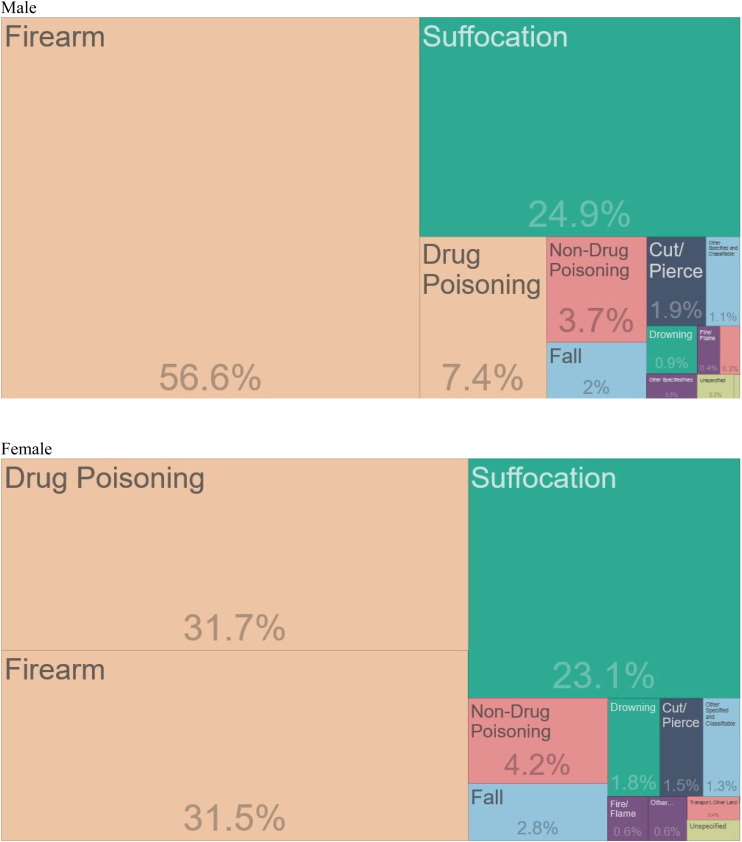
Methods of suicide in the US from 2001 to 2018, by sex^2^.

Other agents affecting the lethality of a suicide attempt include the method, which could be considered a vector in classic infectious disease epidemiology. Figure 3 shows the method of suicide for all US suicides from 2001 to 2018, by sex. Among men, firearms are used in 56.6% of suicide deaths; self-inflicted gunshot wounds are highly lethal (Fowler, Dahlberg, Haileyesus, & Annest, 2015; Miller & Hemenway, 2008; Miller, Barber, White, & Azrael, 2013), whereas intentional overdoses are much more likely to be non-fatal (Miller & Hemenway, 2008). Suffocation (e.g. hanging) is the next most commonly used lethal method of suicide among men, accounting for a quarter of deaths. Among women, there is an approximately equal percentage of suicide deaths attributable to firearms (31.5%) and drug poisoning (31.7%), followed by suffocation (23.1%).

## Host factors that are associated with suicide risk

Much of the literature in suicide epidemiology has covered the host, or individual-level factors, that increase risk (Franklin et al., 2017). Demographic factors, including age, race, socio-economic status are among the most well-replicated and strong risk factors for suicide death (Martínez-Alés & Keyes, 2019). Several individual-level factors have been the subject of review articles. Indeed, personal or family history of suicide attempts, as well as personal, or family history, or psychiatric disorders (Franklin et al., 2017; Olfson et al., 2017b), especially mood disorders (Olfson et al., 2016), are among the most well-replicated risk factors, including both suicide attempts as well as suicide. However, the vast majority of individuals with a psychiatric disorder, even severe disorders, will not die by suicide (Cavanagh, Carson, Sharpe, & Lawrie, 2003). Beyond mental health, there are well-replicated social and ethnic disparities in suicide epidemiology; individuals with financial stress, employment, and poverty (Goldman-Mellor, 2015), are at increased risk of suicide. Indigenous populations have increased vulnerabilities to psychiatric disorders and depressive symptoms due to generations of persecution, poverty, and poor access to health care. Trauma exposure more generally is associated with increased risk of suicide through increasing risk for psychiatric disorders as well as other mechanisms (Borges et al., 2006; Petruccelli, Davis, & Berman, 2019). Individuals on active duty and veteran military members, for example, have elevated risk of suicide, especially in recent years (Ursano et al., 2015). Other risk factors are unique to some stages of suicidal behavior. For example, women are overrepresented in suicide attempts, whereas men are overrepresented in suicide (Callanan & Davis, 2012), and the motivations underlying suicidal behavior that result in death can differ from those that do not (Kessler, Berglund, Borges, Nock, & Wang, 2005). Risk factors vary in association with various stages of suicidality, including birth month (Elbogen et al., 2020). The ways in which these factors influence suicide risk are dynamic across time and the life course; factors may increase baseline vulnerability to intense periods of suicidality, as well as serve as triggers for suicidal episodes.

## Environmental factors that increase suicide risk

Many communicable diseases are influenced by environmental factors. For suicide, these may include not only geography and climate, but also social contexts, such as social network patterns and the macro-economic conditions of a geographic area. In the US, meta-analytic estimates suggest that firearm access, for example, increases the risk of suicide death between two and three-fold (Anglemyer, Horvath, & Rutherford, 2014).

While less proximally dangerous than firearms, other environmental factors that increase suicide rates include altitude (Brenner, Cheng, Clark, & Camargo, 2011), warmer seasons (Woo, Okusaga, & Postolache, 2012), and generally warmer temperatures and areas with more sunshine, although the increase is non-linear and is dependent on other built environmental factors (Kim et al., 2019; Petridou, Papadopoulos, Frangakis, Skalkidou, & Trichopoulos, 2002). Additionally, contextual economic factors such as country-level economic recession and rurality (Norström & Grönqvist, 2015; Oyesanya, Lopez-Morinigo, & Dutta, 2015) have also been implicated. Recessions generally increase the risk for suicide, although results are not uniform (Margerison-Zilko, Goldman-Mellor, Falconi, & Downing, 2016). Home foreclosures predict at least part of the increase in suicide among middle-aged men during and after the recession period (Houle & Light, 2014). Given that the 2020 global economy is experiencing the worst recession since the US depression, monitoring suicide trends is critical.

## Using epidemic modeling approaches to simulate and forecast suicide outbreaks

While not all deaths by suicide arise from communicable processes, the unignorable spatial–temporal correlations indicate that understanding the communicable dynamics of suicide risk serves to deepen the literature on the natural course and etiology of suicide. These processes are not linear, and non-suicidal self-injury as well as non-fatal suicidal behavior often have different underlying mechanisms and risk groups than death by suicide (Fox et al., 2015). Developing mathematical models of suicide dynamics might provide forecasting tools for public health planning and resource allocation as well as clinical risk assessment.

Mathematical models for the infectious disease have come into sharp focus as the COVID-19 pandemic has progressed. Much like with other infectious disease outbreaks, these models are used to: (1) understand transmission processes and forecast geographic spread during the emergence of novel pathogens, including COVID-19 but also recently for Chikungunya, Zika (Reis & Shaman, 2018), pandemic flu (Yang, Lipsitch, & Shaman, 2015), and Ebola (Backer & Wallinga, 2016); (2) assess the severity and activity of recurrent infectious outbreaks, such as for malaria (Smith et al., 2012), seasonal flu (Biggerstaff et al., 2016, 2018), and West Nile virus (DeFelice et al., 2018); and (3) guide and evaluate prevention and interventions, such as for HIV (Sucharitakul, Boily, Dimitrov, & Mitchell, 2018). For suicide, an analogous set of forecasting models would enable better tracking of suicide activity, identification of clustering of suicidal risk, evaluation and quantification of social and environmental drivers of clustering, prediction of future suicidal behaviors, and more effective and cost-efficient targeting of interventions.

Communicable diseases vary in host, agent, and environmental properties. These differences influence disease transmission and occurrence of epidemics, which are further affected by modes of transmission, severity of symptoms, and natural course. Yet all communicable diseases can be described in part by their basic reproductive number, *R*_0_, which is defined as the number of secondary infections caused by a single infectious person in a fully susceptible population. When *R*_0_ is >1, periodic epidemics of disease are possible, and the rate at which these epidemics propagate depends on population susceptibility to infection, patterns of contact with the infectious source, and duration of exposure.

Given this established paradigm for communicable diseases and the observed spatial–temporal clustering of suicide consistent with contagious spread, several fundamental questions need to be addressed: What is the basic reproductive number of suicidal behavior? Does it differ across developmental periods, gender, environment and location, and for various stages of suicidal behavior, including contemplation, ideation, plan, attempt, and death? Suicide causation unfurls over a life course of accumulating risk factors, from distal childhood experiences to acute events that are associated with imminent death. If we can quantify the extent to which suicide and suicide attempts are transmitted within and across populations, heterogeneity in this transmission by imminent risk factors *v.* distal, and elucidate mechanisms by which this transmission is borne, this understanding can help guide control efforts.

Mathematical modeling approaches have been used to address many similar questions for infectious diseases. Models are used to simulate observed outbreaks, identify critical social and environmental drivers of disease, infer key epidemiological features (e.g. *R*_0_), and develop and test appropriate interventions (e.g. school closures, vaccine deployment). Similar to prevention strategies such as facemasks and distancing to slow the COVID-19 pandemic, suicide prevention efforts such as prevention of lethal means and the promotion of social connections may be critical to slow suicide epidemics, and can be modeled. These dynamic models can provide calibrated, real-time ensemble forecasts of future outcomes, much as numerical weather models generate weather forecasts. Infectious disease forecasting has advanced markedly over the past decade and is now routinely used by health agencies to plan for and respond to disease outbreaks (Centers for Disease Control and Prevention, 2020a). Importantly, incomplete ascertainment of cases, which has been cited as a principal limitation of understanding suicide in the US (Bohnert et al., 2013), also impedes infectious disease surveillance. However, detecting signals through noisy, partially-observed data is a central feature of infectious disease modeling and forecasting.

## Mathematical models to predict and forecast suicide are underutilized, but evidence supports their validity and utility

Previous studies have used quantitative models to understand risk factors for and dynamics of suicide (Belsher et al., 2019; Kessler et al., 2017; Torous et al., 2018), suggesting the amenability of these models to assessing suicide risk and evaluating potential points of intervention. Machine learning and other prediction-focused models, which do not simulate processes, but nonetheless use computational algorithms to identify data-driven patterns, are increasingly common in suicide research (Belsher et al., 2019). However, the results have not always been promising. A recent meta-analysis and prediction model incorporating 17 existing prediction models from cohort data demonstrated that although the models themselves have internal accuracy and reliability, they are, in totality, currently relatively poor vehicles for prospectively identifying future suicide events (Belsher et al., 2019). Positive predictive values from these efforts remain frustratingly low, reinforcing the common thread that suicide is difficult to predict accurately, yet the difficulty does not preclude the necessity. Positive predictive values from these efforts remain frustratingly low, reinforcing the common thread that suicide is difficult to predict accurately, yet the difficulty does not preclude the necessity. The absence of data on both distal risk factors that increase vulnerability to suicide and suicide attempts as well as proximal data that predict imminent suicide death remain a challenge impeding the development of more sophisticated and accurate prediction models. However, technology is advancing, and data sources are rapidly expanding (Kessler, Bossarte, Luedtke, Zaslavsky, & Zubizarreta, 2020).

Other computational approaches for estimating and forecasting suicide risk have focused on Markov chain, compartmental, and agent-based models. These models can account for the dynamic transitions between states of the suicidal process, from suicidal ideation, to attempt, to death (Keyes, Hamilton, Swanson, Tracy, & Cerdá, 2019; Mesoudi, 2009). These models are diverse in the research questions asked and parameterizations used (Yip, So, Kawachi, & Zhang, 2014), illustrating quantitatively the added public health impact of population-based approaches. Agent-based models focusing on the transmissibility of suicidal behavior through homophily and social learning have demonstrated that specific dynamics can generate anomalous increases in rates after, for example, the suicide of a celebrity (Mesoudi, 2009). These simulation approaches acknowledge and provide bounds for the transmissibility of suicidal behavior via person-to-person and point-source outbreak mechanisms.

## Available suicide data pose challenges and opportunities for mathematical modeling

Methods to model and forecast suicide as a contagious process are capable of representing the dynamics and handling of data recording issues specific to suicide. Specifically, mathematical models can be tailored to represent key features of suicide epidemiology and can flexibly work with misclassification, biases, and gaps associated with suicide observations.

For many diseases, the risk factors for non-fatal *v.* fatal outcomes depend on the virulence and pathogenicity of the agent, as well as demographic risk factors. For suicide, there are several risk factors that distinguish non-fatal and fatal suicidal behavior, such as sex (Callanan & Davis, 2012), and suicide method (Fowler et al., 2015; Gunnell, Bennewith, Hawton, Simkin, & Kapur, 2005). Further, local suicide clusters are primarily limited to adolescents and young adults (Gould et al., 1990; Johansson, Lindqvist, & Eriksson, 2006; Joiner, 1999), underscoring the importance of separately modeling different developmental stages. Increases in suicide after the death of Robin Williams, in contrast primarily affected middle-aged men (Fink et al., 2018). Suicide is not unique, as many communicable diseases are also strongly patterned by age, and models have been well-developed to represent age-related variation. Further, suicide is an outcome that is strongly determined from within-person change, sometimes over a short period of time as an individual enters a suicidal crisis. Existing agent-based models to examine suicide risk factors (Keyes et al., 2019) are highly tuned to such individual changes, and can simultaneously model within-person mood and agitation changes, using within-period data for calibration, with between-person risk factor distributions.

Mathematical models of communicable diseases regularly handle variations in the lethality of agents. For example, seasonal influenza has 3–4 strains circulating in any particular year, some of which are relatively mild and others that have increased risk of serious complications and death. Identifying the lethality of agent strains and incorporating these features into the model building is thus another strength of the mathematical modeling approach. Further, by including variation in the dynamics of suicide by the method of lethality, it may be possible to improve projections when the availability of an attempt method changes. For example, changes to firearm availability affect suicide rates (Brent et al., 1991; Kposowa, Hamilton, & Wang, 2016; Miller et al., 2013), and simulations can incorporate these changing dynamics in mathematical models.

A unique feature of suicide is that designation of death by suicide requires subjective assessment regarding intentionality of the act. While in some cases the intention is clear (e.g. when a suicide note is recovered), other deaths are more difficult to adjudicate, and often survivors of serious suicide attempt even report that their level of intentionality was not clear even to them. For example, in 2016, drug poisoning was used in an estimated 14.7% of suicide deaths (Stone et al., 2018), but certification of ‘unintentional’ drug poisoning deaths (especially using opioids) is much more common even when the intentionality may be ambiguous (Maloney, Degenhardt, Darke, & Nelson, 2009). Certification practices for determining intentionality may also differ across states and jurisdictions.

Further, most individuals who contemplate or attempt suicide do not come to clinical attention (Centers for Disease Control and Prevention, 2018b; Olfson et al., 2017a) and thus are not captured in clinical databases. Community samples estimate the annual prevalence of suicide attempts is ~7% among adolescents and 0.8% among adults (Olfson et al., 2017a). Yet estimated rates of suicide attempts in hospital settings is 0.32% among adolescents and 0.15% among adults (Centers for Disease Control and Prevention, 2020b), suggesting that population rates of suicide attempts based on hospital records may be underestimated 10–20-fold, especially for less medically severe attempts. However, these data gaps are not unique or particularly problematic compared to other health outcomes with informative modeled systems. For example, an estimated 90% of influenza cases do not come to clinical attention, yet mathematical systems for predicting flu are sophisticated, accurate, and useful. Further, data on suicidal behavior are quite well-characterized compared to many diseases for which mathematical models are applied. Nevertheless, significant data gaps remain. Warning signs for suicide (e.g. agitation, making a plan, acute life events) provide critical information for prediction, yet few data sources are available to capture such information, which also hinders progress.

## Novel sources of surveillance hold promise to augment existing sources

A central challenge to mathematical modeling, and to the understanding of health processes in general, is the quality and completeness of surveillance data sources, as well as the timeliness of data. Just as in most areas of epidemiological surveillance, there is incomplete ascertainment of suicidal behavior in data capture systems, and misclassification. Furthermore, hospitalization and death data are often released months or years after occurrences, making real-time or near real-time assessments difficult. Yet novel sources of data are continuously being developed and evaluated for validation of suicidal behavior, with substantial potential.

Novel sources of data collection for self-harm and suicide includes social media and search term analyses. With billions of social media users and daily internet searches, social media and search terms have emerged as a reliable albeit noisy indicator of population mental health, including suicidal behavior (Homan, Johar, Liu, SIlenzio, & Ovesdotter Alm, 2014; Reece et al., 2017). For example, Eichstaedt et al. (2018) established that Facebook status updates predict medical-record-documented depression up to three months before diagnosis with fair accuracy (Eichstaedt et al., 2018). Online sources such as Twitter (Fahey, Boo, & Ueda, 2020; Roy et al., 2020) and Reddit (De Choudhury, Kiciman, Dredze, Coppersmith, & Kumar, 2016) have been leveraged for content and shifts in writing style, word choice, and other natural language indicators that could serve as sentinel signals of an emerging suicidal crisis, and could be used in epidemiological surveillance moving forward as the field and methods become more sophisticated. However, concerns about intrusion and privacy require careful ethical assessment as this field moves forward.

The most well-researched area of novel data collection is Google. More than 20 studies across the world have examined Google search terms related to suicide as a potential indicator of suicidal behavior as reviewed by Lee, 2020; Tran et al., 2017. These studies have documented some evidence for face-validity of search terms as data collection tools, including that Google search terms related to depression and suicide exhibit seasonal variation that is similar in correlation to seasonal variation in recorded depression and suicide. In terms of correlation with suicidal behavior, there is substantial heterogeneity in the strength of the relationship. Tran et al. (2017) documented that many Google search terms were too broad to reliably capture underlying signals of suicidal behavior, but that validity improves with search terms for more specific behaviors, indicative of a trade-off between sensitivity and specificity (Tran et al., 2017).

**Fig. 4.**
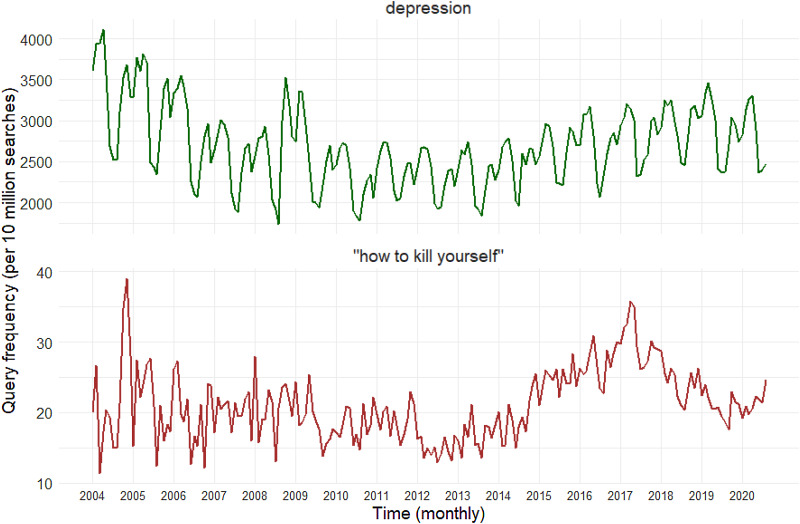
US National Google search trends for suicide-related terms (‘depression’, and ‘how to kill yourself’) from January 2004 to August 2020.

Figure 4 documents the query frequency (Zepecki, Guendelman, DeNero, & Prata, 2020) of the US Google searches for ‘depression’ and ‘how to kill yourself’, two search terms that have been demonstrated to capture mental health and suicidal behavior, from January 2004 to August 2020. Searches for ‘depression’ exhibit a seasonal trend, with annual increases in winter months and decreases in summer months, consistent with known seasonality of depression diagnoses, providing face validity. Further, searches for ‘how to kill yourself’, while less seasonal, exhibit a clear upward trend since approximately 2010–2011, which is consistent with the time period in which suicide deaths exhibited sharp upward trends.

## Summary

Suicide in the US continues to be an urgent public health issue. The collateral effects of the COVID-19 pandemic (e.g. economic hardship, social disruption) will unfold in ways that will likely accelerate these increases, especially among the most vulnerable populations (Reger, Stanley, & Joiner, 2020). Suicide shares many processes and underlying dynamics with communicable diseases and should be studied with the same tools. This begins with basic epidemiological activities such as estimating the reproductive rate and modeling the dynamics of transmission in ways that enable inferences about who, when and where outbreaks will occur. Robust suicide surveillance information, coupled with new and novel sources of data provide a solid foundation for such models. The results could help anticipate and focus screening and other preventative efforts on emerging high-risk populations and thus deter additional outbreaks from occurring.

## Notes

1 Data are from the Multiple Cause of Death Files, 1999–2018, as compiled from data provided by the 57 vital statistics jurisdictions through the Vital Statistics Cooperative Program, National Center for Health Statistics, Centers for Disease Control and Prevention. Intentional self-harm ICD-10 codes: U03, X60-84, Y87.0. Accessed at http://wonder.cdc.gov/ucd-icd10.html on Apr 22, 2020.

2 Source: Deaths data from National Center for Health Statistics. Visualization: Web-based Injury Statistics Query and Reporting System (WISQARS) Fatal Injury Data Visualization Tool. Accessed at: https://wisqars-viz.cdc.gov:8006/explore-data/home on April 22, 2020.
